# The BET inhibitor/degrader ARV-825 prolongs the growth arrest response to Fulvestrant + Palbociclib and suppresses proliferative recovery in ER-positive breast cancer

**DOI:** 10.3389/fonc.2022.966441

**Published:** 2023-01-18

**Authors:** Ryan M. Finnegan, Ahmed M. Elshazly, Nipa H. Patel, Liliya Tyutyunyk-Massey, Tammy H. Tran, Vishnu Kumarasamy, Erik S. Knudsen, David A. Gewirtz

**Affiliations:** ^1^ Departments of Microbiology & Immunology, Virginia Commonwealth University, Richmond, VA, United States; ^2^ Departments of Pharmacology & Toxicology, Virginia Commonwealth University, Richmond, VA, United States; ^3^ Massey Cancer Center, Virginia Commonwealth University, Richmond, VA, United States; ^4^ Department of Pharmacology and Toxicology, Faculty of Pharmacy, Kafrelsheikh University, Kafrelsheikh, Egypt; ^5^ Department of Molecular and Cellular Biology, Roswell Park Comprehensive Cancer Center, Buffalo, NY, United States

**Keywords:** cancer, autophagy, senescence, fulvestrant, Palbociclib, ARV-825

## Abstract

Anti-estrogens or aromatase inhibitors in combination with cyclin-dependent kinase 4 and 6 (CDK4/6) inhibitors are the current standard of care for estrogen receptor-positive (ER+) Her-2 negative metastatic breast cancer. Although these combination therapies prolong progression-free survival compared to endocrine therapy alone, the growth-arrested state of residual tumor cells is clearly transient. Tumor cells that escape what might be considered a dormant or quiescent state and regain proliferative capacity often acquire resistance to further therapies. Our studies are based upon the observation that breast tumor cells arrested by Fulvestrant + Palbociclib enter into states of both autophagy and senescence from which a subpopulation ultimately escapes, potentially contributing to recurrent disease. Autophagy inhibition utilizing pharmacologic or genetic approaches only moderately enhanced the response to Fulvestrant + Palbociclib in ER+ MCF-7 breast tumor cells, slightly delaying proliferative recovery. In contrast, the BET inhibitor/degrader, ARV-825, prolonged the growth arrested state in both p53 wild type MCF-7 cells and p53 mutant T-47D cells and significantly delayed proliferative recovery. In addition, ARV-825 added after the Fulvestrant + Palbociclib combination promoted apoptosis and demonstrated efficacy in resistant RB deficient cell lines. These studies indicate that administration of BET inhibitors/degraders, which are currently being investigated in multiple clinical trials, may potentially improve standard of care therapy in metastatic ER+ breast cancer patients and may further prolong progression-free survival.

## 1 Introduction

The number of new cases of invasive breast cancer in women is projected to be over 287,000 in 2022, resulting in over 43,000 deaths; in fact, rates of breast cancer have been gradually increasing by 0.5% per year since the mid-2000s (1). Amongst these cases, approximately 73% are comprised of hormone-receptor–positive breast cancer (2). The first line hormonal therapy for this subtype of breast cancer utilizes selective estrogen receptor modulators (SERMs) such as tamoxifen, aromatase inhibitors (AIs), and selective estrogen receptor degraders (SERDs), such as Fulvestrant (3). Tamoxifen, a SERM that has been utilized in the clinic for decades, is usually prescribed to treat hormone receptor positive, early-stage breast cancer after surgery to reduce disease recurrence. Despite reducing recurrence for up to five years, resistance to hormonal therapy is a major drawback to the success of these therapies and most cases eventually result in metastatic disease progression (4, 5).

The current standard of care for metastatic ER-positive/Her2 negative breast cancer utilizes the combination of either the estrogen receptor degrader Fulvestrant or aromatase inhibitors such as letrozole with CDK4/6 inhibitors such as Palbociclib (6, 7). Fulvestrant binds and destabilizes the estrogen receptor, resulting in destruction of the receptor by the cells’ normal degradation pathways (8). CDK 4/6 inhibitors, such as Palbociclib, interfere with cell cycle progression by suppressing the CDK-cyclin complex mediated phosphorylation of Rb, thereby allowing the dephosphorylated form of Rb to bind to the transcriptional regulator, E2F, blocking G1/S cell cycle transition (7, 9). In patients whose disease has progressed on prior endocrine therapy, the combination of Fulvestrant with Palbociclib had extended progression-free survival in breast cancer patients from 4.6 to 11.2 months (10). While these treatments represent remarkable improvements, escape from the tumor suppressive effects of even the combination therapies occurs frequently, with the consequence that the patients unfortunately succumb to this disease.

Disease recurrence in breast cancer has been linked to escape from tumor dormancy. Senescence, originally thought to be permanent form of growth arrest, is now recognized as a transient arrest that could be a form of tumor dormancy (11, 12). Senescent tumor cells secrete tumor promoting factors, which has led to preclinical studies of the capacity of senolytics for their elimination. Our laboratory, along with others, have reported on the promotion and senescence by cancer therapies as well as clearance of senescent tumor cell populations by senolytic agents (12–16). Several senolytic agents such as BCl_2_/BCL-xL inhibitors Navitoclax (ABT-263), and Venatoclax (ABT-199), Panobinostat and Fisetin are currently in preclinical studies and/or in clinical trials with (17–20).

Almost invariably, autophagy also accompanies the induction of senescence by chemotherapy and radiation (21). Autophagy or “self-eating” is an evolutionarily conserved catabolic process through which cellular cargo is sequestered within a double membrane vesicle and ultimately undergoes lysosomal degradation. Many antitumor therapies, such as chemotherapy and radiation, have been shown to promote the cytoprotective form of autophagy, whereby autophagy inhibition sensitizes the tumors to the therapy (22, 23). It is well established in the literature that endocrine therapies display protective autophagy and consequently the autophagy inhibitors chloroquine and hydroxychloroquine show positive outcomes in combination with endocrine therapies, such as fulvestrant or tamoxifen, in preclinical studies (24). Additional studies have investigated the induction of autophagy by palbociclib and the impact of autophagy inhibition on drug sensitivity in breast tumor cells (25). These studies, however, did not involve the current standard of care combination of fulvestrant + Palbociclib.

In general, studies have focused on either autophagy or senescence as separate and distinct responses which might be manipulated for therapeutic benefit. However, autophagy and senescence often occur in parallel in response to cancer therapy (21, 26). In the current work, we demonstrate that Palbociclib and Fulvestrant promote both autophagy and senescence, suggesting that either inhibition of autophagy or the addition of senolytics might enhance and/or prolong the therapeutic response. However, neither pharmacologic inhibition of autophagy nor genetic silencing of the autophagy regulatory gene, ATG5, produced more than a modest improvement in the response to Fulvestrant + Palbociclib, particularly with respect to proliferative recovery. In contrast to the relative lack of impact of autophagy inhibition, the BET degrader, ARV-825, significantly prolonged growth arrest and delayed proliferative recovery in both p53 wildtype MCF-7 cells and p53 mutant T-47D cells independent of Rb status. Consistent with recent studies utilizing BET inhibition (27), these observations suggest that the utilization of BET inhibitors/degraders in sequence with Fulvestrant + Palbociclib may provide a therapeutic advantage for breast cancer patients undergoing standard of care therapy.

## 2 Methods

### 2.1 Cell lines

MCF7 cells were generously gifted by Dr. Charles Clevenger, at Virginia Commonwealth University. MCF7 and T47D cells were cultured in DMEM medium supplemented with 10% (*v*/*v*) fetal bovine serum (Thermo Scientific, SH30066.03), 100 U/mL penicillin G sodium (Invitrogen, 15140–122), and 100 μg/mL streptomycin sulfate (Invitrogen, 15140–122).

The ATG5-knockdown was generated as follows: Mission shRNA bacterial stocks of ATG5 were purchased from Sigma Aldrich. Lentivirus was produced in HEK 293T cells, which were co-transfected using EndoFectinTM Lenti Transfection Reagent (GeneCopoeia, 1001-01) with a packaging mixture of psPAX2 and pMD2.G constructs (Addgene). Media containing the virus was used to infect the MCF7 cells. Puromycin (1 μg/ml) was used as a selection marker to enrich for the infected cells.

Lentiviral packaging of H2B-GFP vector, pLenti0.3UbCGWH2BC1-PatGFP was carried out in 293FT cells. Exponentially growing MCF7 and T47D cells were infected with the H2B-GFP lentivirus in the presence of polybrene (4µg/ml). GFP positive cells were selected using FACS Aria cell sorter.

### 2.2 Drug treatment

Fulvestrant (Millipore Sigma, I4409), Palbociclib (LC Laboratories, P-7788), Bafilomycin A1 (Millipore Sigma, 196000), and ARV-825 (MedChemExpress, HY-16954) were dissolved in DMSO. Chloroquine (Millipore Sigma, C6628) was dissolved in sterile 1X PBS (Thermo Fisher Scientific, 10010).

For sensitization studies, cells were exposed to Fulvestrant (100 nM), Palbociclib (1 uM) or the combination for 6 days.

For early autophagy inhibition studies, cells were pre-treated with CQ (10 uM) or Baf (2.5 nM) for 2 h prior to exposure with Fulvestrant, Palbociclib or the combination. CQ or Baf were given in combination with the respective conditions for an addition 48 h, CQ or Baf was removed and Fulvestrant, Palbociclib or the combination were given for an additional 4 days post-CQ or Baf removal to meet the 6-day drug treatment regimen.

For late autophagy inhibition studies, cells were treated with Fulvestrant, Palbociclib or the combination for 6 days and CQ (10 uM) or Baf (2.5 nM) was given for 48 h post-anti-estrogen and CDK 4/6 inhibition therapy.

For senolytic exposure, cells were treated with Fulvestrant, Palbociclib or the combination for 6 days and the respective senolytic was given for 48 h post-anti-estrogen and CDK 4/6 inhibition therapy. ARV-825 was administered for 96 h post-anti-estrogen and CDK 4/6 inhibition therapy.

### 2.3 Cell viability

Trypan blue exclusion was utilized to assess cell viability. Cells were plated at 20,000 cells per well in a 6-well plate and treated with the respective conditions. On the indicated days, cells were trypsinized, stained with 0.4% trypan blue (Sigma, T01282), and counted on the indicated days using a hemocytometer. Growth curves were generated from the collected data.

### 2.4 Clonogenic survival assay

Cells were plated at a density of 200 cells per well in 6-well plates and treated with the respective conditions. Media was replenished every other day until colonies form. Colonies were washed with 1X phosphate-saline buffer (PBS, Life Technologies), fixed with 100% methanol and stained with 0.1% crystal violet (Sigma). The number of colonies formed were counted.

### 2.5 Promotion of apoptosis

The extent of apoptotic cell death was measured using Annexin V-FITC/Propidium iodide staining. On the indicated day, cells were trypsinized, washed with 1X PBS and stained according to manufacturer protocol (Annexin V-FITC Apoptosis Detection Kit; BD Biosciences, 556547). Fluorescence was quantified by flow cytometry using BD FACSCanto II and BD FACSDiva software at the Flow Cytometry Core Facility at Virginia Commonwealth University. For all flow cytometry experiments, 10,000 cells per replicate were analyzed and three replicates for each condition were analyzed per independent experiment unless otherwise stated. All experimental protocols were performed with cells protected from light.

### 2.6 Acridine orange staining

On the indicated days, cells were stained with 1 μg/ml acridine orange at 37°C for 20 min and then washed with 1X PBS. Cells were imaged using an inverted fluorescence microscope (Olympus, Tokyo, Japan) at 20X magnification. For quantification of autophagic vesicles (AVOs), on the indicated days, cells were trypsinized, harvested and washed with 1X PBS. Pellet fractions were resuspended in 1X PBS and analyzed by BD FACSCanto II and BD FACSDiva software. For all flow cytometry experiments, 10,000 cells per replicate were analyzed and three replicates for each condition were analyzed per independent experiment unless otherwise stated. All experimental protocols were performed with cells protected from light.

### 2.7 SA-ß-gal staining

On the indicated days, cells were stained with X-gal (5-bromo-4-chloro-3-indolyl-β-D-galactopyranoside) staining as previously described by Dimri et al. (28). Cells were washed with 1X PBS and phase contrast images were taken using an inverted microscope (Olympus, Tokyo, Japan).

T47D-WT and Rb-deleted cells were treated with Palbociclib (1 µM) in combination with Fulvestrant (100 nM) for 6 days. The cells were stained for β-galactosidase to determine the senescence phenotype by using the commercially available kit (Cell Signaling; 9860) according to the manufacturer’s protocol. Cell images were taken using phase-contrast microscope at 20X magnification.

To quantify ß-gal positive senescent cells, after treatment, cells were treated with Bafilomycin A1 (100 nM) for 1 h to achieve lysosomal alkalinization, followed by staining with C_12_FDG (10 μM) for 2 h at 37 °C. After incubation, cells were collected and analyzed by BD FACSCanto II and BD FACSDiva software. For all flow cytometry experiments, 10,000 cells per replicate were analyzed and three replicates for each condition were analyzed per independent experiment unless otherwise stated. All experimental protocols were performed with cells protected from light.

### 2.8 Western blot analysis

Western blotting was performed as previously described (29). In brief, after indicated treatments, cells were trypsinized, harvested, and washed with 1X PBS. Pellets were lysed and protein concentrations were determined by the Bradford Assay (Bio-Rad Laboratories, 5000205). Protein samples were loaded and subjected to SDS-polyacrylamide gel electrophoresis, transferred to polyvinylidene difluoride membrane, and blocked with 5% milk in 1X PBS with 0.1% Tween 20 (Fisher, BP337). The following primary antibodies were used: SQSTM1/p62 (Cell Signaling Technology, 5114T); c-Myc (Cell Signaling Technology, 5605); ATG5 (Cell Signaling Technology, 2630); LC3B (Cell Signaling Technology, 3868); BRD4 (Cell Signaling Technology, 13440S); B-actin (Cell Signaling Technology, 4970); and GAPDH (Cell Signaling Technology, 2118). The membrane was incubated overnight at 4°C with the indicated primary antibodies at a dilution of 1:1000 in 5% BSA. Secondary antibodies: Horseradish peroxidase (HRP)-conjugated secondary antibodies (Cell Signaling, anti-mouse, 7076S; anti-rabbit, 7074S). The membrane was then washed, secondary antibody was added at a dilution of 1:2000 in 5% BSA for 2 h at room, and the membrane was washed again with 1X PBS with 0.1% Tween 20 three times. Blots were developed using Pierce enhanced chemiluminescence reagents (Thermo Scientific, 32132) on BioRad ChemiDoc System.

### 2.9 qRT-PCR

Fulvestrant + Palbociclib treated cells were harvested at Day 6 after drug treatment, and total RNA was extracted using RNeasy kit (QIAGEN, Germany) following the manufacturer’s instructions. cDNA was synthesized using iScript cDNA Synthesis Kit (BioRad, USA) based on the protocol that manufacture provided. cDNAs from different samples were amplified in technical triplicates using iTaq Universal SYBR^®^ Green Supermix from BioRad in QuantStudio™ 3 Real-Time PCR System (Thermofisher, USA). QuantiTect primers were purchased from Qiagen: CXCL8: QT0000322; IL-6: QT00083720; IL-1*β*: QT00021385; MMP3: QT00060025; GAPDH: QT00079247. Relative mRNA expression was determined using the ΔΔCt method.

### 2.10 Cell proliferation assay

To determine the proliferation of T47D WT and Rb-deficient cells in real time, live cell imaging using IncuCyte S3 was performed. Cells were seeded in 96 well dish (1000 cells/well) and allowed to adhere overnight. The cells were exposed to Palbociclib (1 µM) in combination with Fulvestrant (100 nM) for 6 days and the cell division as monitored using IncuCyte that performs nuclei count. Following 6 days, the cells were released from the Palbociclib/Fulvestrant combination treatment and allowed to grow in the absence and presence of ARV825 (50 nM) for 96 H. Following 96H exposure, the cells were released from ARV825, and the cellular outgrowth was monitored. Based on the nuclei counts, the relative proliferation rate was determined. Growth cures were generated using GraphPad Prism.

### 2.11 Statistics

Unless otherwise indicated, all quantitative data is shown as mean ± SEM from at least three independent experiments, all of which were conducted in triplicates or duplicates. GraphPad Prism 9.0 software was used for statistical analysis. All data was analyzed using either a one- or two-way ANOVA, as appropriate, with Tukey or Sidak *post hoc*.3 Results

## 3 Results

### 3.1 The growth arrest response to Fulvestrant + Palbociclib in MCF7 breast tumor cells

**Figure 1:**
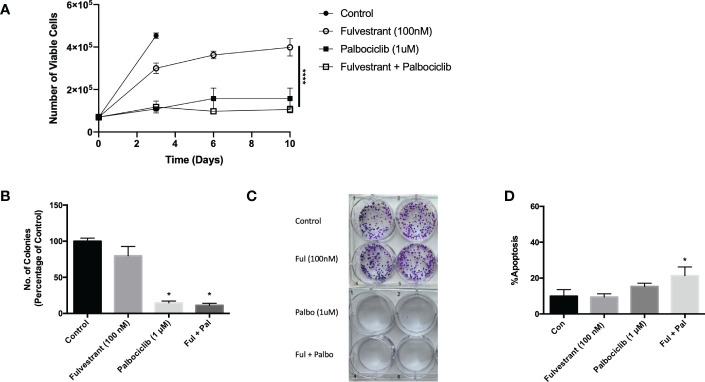
Fulvestrant in combination with Palbociclib sensitizes MCF7 cells. Cells were treated with Fulvestrant (100 nM), Palbociclib (1 µM) or the combination for 6 days **(A-C)**. **(A)** Viability of MCF7 cells was monitored based on trypan blue exclusion at indicated days following drug exposure (n=3). ****P ≤ 0.0001 indicate statistical significance of each condition compared to Fulvestrant alone. **(B)** After exposure for 6 days, cells were incubated in fresh medium for 7 days. Quantification of colonies expressed as relative percentage compared to controls (n=3). **(C)** Representative colony formation 7 days after drug removal by crystal violet staining. **(D)** Apoptosis was measured using annexin V/PI staining at the end of the 6-day treatment and fluorescence was measured using flow cytometry (n=3). Unless stated otherwise, data were from three independent experiments. *P ≤ 0.05 indicate statistical significance of each condition compared to control as determined using two‐way ANOVA with Sidak's post hoc test.

While anti-estrogen therapy combined with CDK 4/6 inhibition is the standard of care for HR+/HER2- breast cancer patients diagnosed with locally advanced or metastatic disease progression following endocrine therapy, this combination therapy only modestly prolongs patient survival (30). In an effort to simulate the clinical treatment regimen in an *in vitro* environment, MCF7 cells were exposed to Fulvestrant and Palbociclib for 6 days and fresh media was replenished after drug removal on day 6. Cell viability was assessed utilizing trypan blue exclusion on the indicated days **(** **Figure 1A**). Fulvestrant initially delayed tumor cell growth and, after a delay, arrested the cells. Palbociclib, alone, and in combination with Fulvestrant, completely halted the growth of the MCF7 cells; however, the cells generally began to recover from treatment after a period of approximately 8-12 days (see **Figures 3**–**6**), which is consistent with earlier work from this and other laboratories (11–13).

The effectiveness of Palbociclib alone as well as the combination treatment in suppressing cell growth was confirmed by clonogenic survival studies (**Figures 1B, C**). The growth inhibitory effect of fulvestrant alone did not achieve significance, in contrast to the moderate effects in the temporal response assay; consequently, the observed effects are largely palbociclib driven. While therapy-induced tumor cell death is the desired outcome of anti-cancer treatment, there was a relatively low degree of apoptosis in MCF7 cells treated with Fulvestrant, Palbociclib or the combination (**Figure 1D**), which may be permissive for proliferative recovery. Consequently, one of the primary goals of the present work was to identify a strategy that might convert the growth arrest response to one of cell death, initially through efforts to block autophagy.

### 3.2 Fulvestrant and Palbociclib-induced autophagy

**Figure 2:**
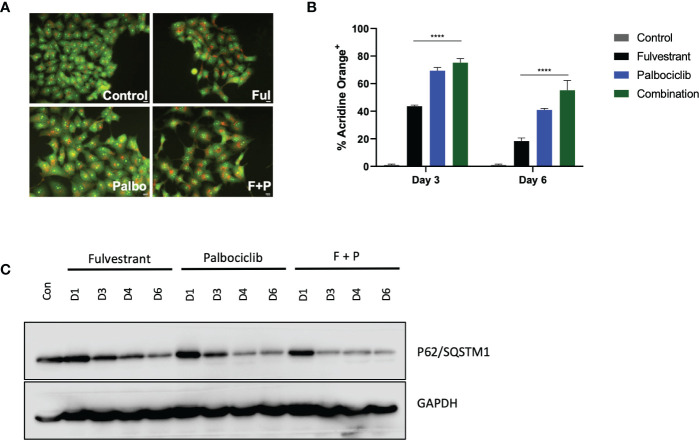
Fulvestrant in combination with Palbociclib induces autophagy. Cells were treated with Fulvestrant (100 nM), Palbociclib (1 µM) or the combination for 6 days **(A–C)**. Lysates were collected at specified days **(A)** Cells were stained with acridine orange on day 4 and imaged using a fluorescent microscope. All images were taken at the same magnification (scale bar= 200 µm, n=3). **(B)** Cells were stained with acridine orange and fluorescence was quantified using flow cytometry. **(C)** Autophagy induction over time was assessed by degradation of p62/SQSTM1 protein levels. All images are representative fields or blots from at least three independent experiments. ****P ≤ 0.001 indicate statistical significance of each condition compared to Fulvestrant as determined using two-way ANOVA with Sidak's *post hoc* test.

In response to therapy, cancer cells upregulate multiple mechanisms in attempts to evade cell death, one of which is autophagy (31, 32). Autophagy is conventionally considered to be a cytoprotective process that allows cells to combat either intrinsic or extrinsic forms of injury; however, other functions of autophagy have been identified, specifically a cytotoxic form (33) and what has been termed as a nonprotective form (31, 34, 35). Consequently, we examined whether autophagy was induced in response to the anti-estrogen, Fulvestrant, and CDK4/6 inhibition therapy in MCF7 cells. Initially, acridine orange was utilized at day 4 to assess acidic vesicle formation. **Figure 2A** demonstrates basal autophagy in these cells as well as increased acidic vesicle generation in response to Fulvestrant or/and Palbociclib. **Figure 2B** further provides quantification of fluorescence *via* flow cytometry and indicates that autophagy is marginally higher for the combination treatment at day 3 and significantly higher at day 6 (although autophagy is reduced overall). To verify autophagy induction, western blot analysis in **Figure 2C** revealed a temporal decline in p62/SQSTM1 levels in MCF7 cells treated with Fulvestrant, Palbociclib and the drug combination.

### 3.3 Efforts to sensitize MCF-7 breast tumor cells *via* autophagy inhibition

**Figure 3:**
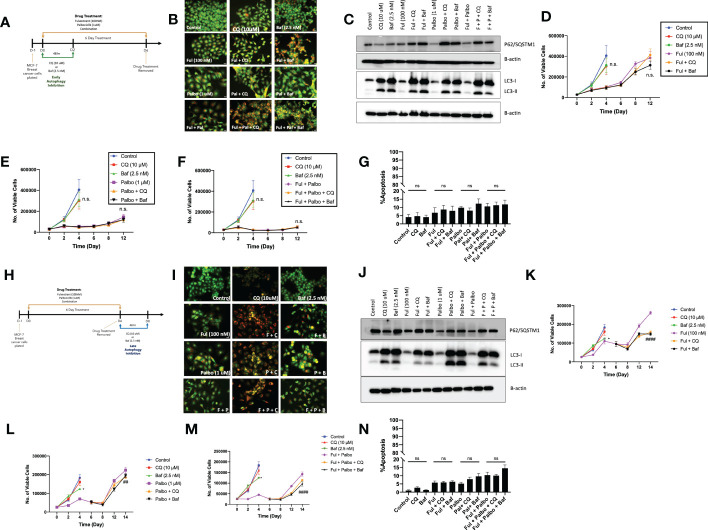
Autophagy inhibition does not alter sensitivity to Fulvestrant in combination with Palbociclib in MCF7 cells. Cells were pre-treated for 3 h with either CQ (10 uM) or Baf A1 (2.5 nM). CQ and Baf A1 were given for an additional 48 h, alongside the treatment with Fulvestrant (100 nM), Palbociclib (1 µM) or the combination for 6 days. **(A)** Schematic of *in vitro* treatment. **(B)** Cells were stained with acridine orange on day 3 and imaged using a fluorescent microscope. All images were taken at the same magnification (scale bar= 200 µm, n=3). **(C)** Western blot analysis at day 3 assessing accumulation of LC3 I-II and p62/SQSTM1 protein levels. **(D–F)** Viable cell number was counted via trypan blue exclusion on the indicated days. **(G)** Apoptosis was measured by annexin V/PI staining. Staining was performed on day 6 and fluorescence was measured using flow cytometry. **(H)** Schematic of in vitro treatment. Cells were treated with Fulvestrant (100 nM), Palbociclib (1 µM) or the combination for 6 days, drugs were removed, and cells were given an additional 48 h CQ (10 uM) or Baf A1 (2.5 nM). **(I)** Cells were stained with acridine orange on day 8 and imaged using a fluorescent microscope. All images were taken at the same magnification (scale bar= 200 µm, n=3). **(J)** Autophagy inhibition was confirmed by western blot analysis assessing accumulation of LC3 I-II and p62/SQSTM1 protein levels. **(K–M)** Viable cell number was counted *via* trypan blue exclusion on the indicated days. **(N)** Apoptosis was measured using annexin V/PI staining. Staining was performed on day 8 and fluorescence was measured using flow cytometry. All images are representative fields, blots, or data from at least three independent experiments. *P ≤ 0.05, ##P ≤ 0.01, and ###P ≤ 0.001, ns (not significant) indicate statistical significance of each condition compared to Fulvestrant, Palbociclib or the combination of Fulvestrant and Palbociclib as determined using two-way ANOVA with Sidak's *post hoc* test.

In an effort to sensitize MCF7 cells to the Fulvestrant, Palbociclib, and the combination therapy, the lysosomotropic agents, chloroquine (CQ) and bafilomycin A1 (Baf), were utilized as pharmacological inhibitors of autophagy, based on their ability to interfere with autophagosome-lysosome fusion (36). Chloroquine and bafilomycin were added concurrently with the Fulvestrant + Palbociclib for 48 hrs; CQ and Baf were removed, and the Fulvestrant + Palbociclib treatment restored for an additional 4 days (**Figure 3A**). Autophagy was visualized by acridine orange staining; acridine orange fluoresces bright orange in acidic environments, such as the lysosomes, and shifts to an orange/yellow color when pH becomes less acidic (37, 38). We also observed an increased number of autophagic vacuoles upon the addition of the CQ and Baf, which is indicative of autophagic vacuole accumulation when the autophagic process is prevented from going to completion (**Figure 3B** **)** (39). The inhibition of autophagy in cells exposed to CQ and Baf was further confirmed by western blot analysis of LC3-II and p62/SQSTM1. We observed an increase in accumulation of LC3-II in the presence of CQ and Baf (due to inhibition of autolysosome formation as well as accumulation of autophagosomes) in both control and drug treated groups, indicating that CQ and Baf inhibited both basal and treatment-induced autophagy in MCF7 and T47D cells (**Figures 3C**, **S1C**). Accumulation of p62/SQSTM1 indicative of interference with p62/SQSTM1 degradation further confirmed that CQ and Baf inhibited treatment-induced autophagy. However, autophagy inhibition failed to sensitize the MCF-7 and T47D cells to Fulvestrant, Palbociclib or the combination therapy (**Figures 3D–F**, **S1B**), indicating that the autophagy was functionally nonprotective, as we have observed previously in other experimental systems (40–42). Of note is the recovery of proliferative capacity that occurs between days 8-12, which is indicative of escape from senescence (see below). Consistent with the lack of sensitization, pharmacological autophagy inhibition did not significantly promote apoptotic cell death by the combination of the anti-estrogen and CDK 4/6 inhibitor **(** **Figure 3G** **).**

Conventionally, most preclinical, and clinical studies designed to evaluate the effect of autophagy inhibition involve the addition of the pharmacological autophagy inhibitors concurrently or as a pre-treatment to therapy (41, 43, 44). Preliminary experiments (not shown) suggested the possibility that a more delayed approach to autophagy inhibition might prove to be a more effective sensitization strategy. Consequently, MCF7 cells were treated with Fulvestrant, Palbociclib or the combination for 6 days, and then exposed to CQ or Baf for 48 hrs post treatment (**Figure 3H**). Similar to the experiments in **Figures 3B, I** demonstrates autophagy inhibition based on interference with lysosomal acidification by acridine orange staining. Autophagy inhibition by Baf and CQ was additionally confirmed by western blot analysis of LC3-II and p62/SQSTM1. Again, similar to the data generated in **Figure 3C**, we observed an increase in LC3-II accumulation (**Figures 3J**, **S1E**) and accumulation of p62/SQTM1 (interference with degradation) with CQ and Baf. **Figures 3K–M** indicate that while addition of CQ or Baf provided a modest degree of sensitization to Fulvestrant alone, in terms of delaying proliferative recovery, sensitization of the MCF7 cells to Palbociclib or the combination treatment was barely significant. Furthermore, no sensitization was observed in T47D cells (**Figure S1E**). Additionally, there was minimal promotion of apoptosis with autophagy inhibition, (**Figure 3N**), again consistent with the autophagy being largely nonprotective in function in this experimental model system.

### 3.4 Efforts to sensitize MCF-7 breast tumor cells by autophagy inhibition *via* genetic silencing

**Figure 4:**
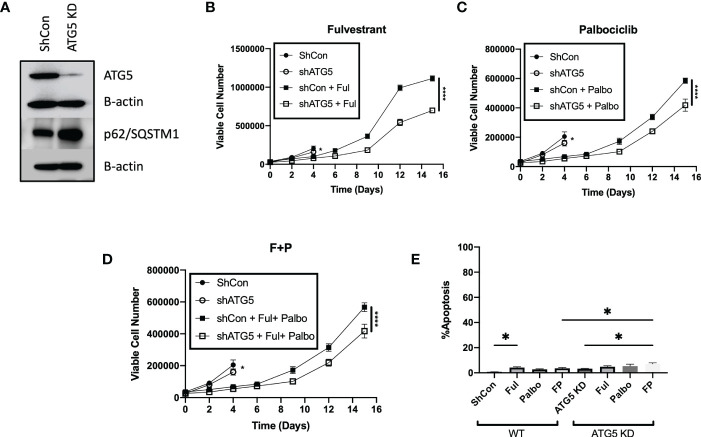
Genetic knockdown of autophagy only moderately increases sensitivity to Fulvestrant in combination with Palbociclib in MCF7 cells. Cells were treated with Fulvestrant (100 nM), Palbociclib (1 µM) or the combination for 6 days **(A–E)**. Short hairpin RNA was used to knockdown ATG5. **(A)** Western blot analysis of ATG5 and p62/SQSTM1 protein levels. **(B–D)** Viable cell number was counted *via* trypan blue exclusion on the indicated days. **(E)** Apoptosis was measured using annexin V/PI staining. Staining was performed on day 8 and fluorescence was measured using flow cytometry. Unless stated, otherwise data were from three independent experiments. *P ≤ 0.05 and ****P ≤ 0.0001 compared to shControl cells treated with Fulvestrant, Palbociclib or the combination of Fulvestrant and Palbociclib.

To further confirm the absence of a pronounced sensitization to Fulvestrant, Palbociclib and the combination treatment *via* autophagy inhibition, the MCF-7 cells were stably transfected using short hairpin RNA for ATG5 (shATG5) or scrambled control (shControl). Knockdown of ATG5 and impairment of autophagy was confirmed by western blot analysis indicating reduced levels of ATG5 and accumulation of p62/SQSTM1 (**Figure 4A**). Temporal analysis of cell viability showed that autophagy deficient cells were significantly more sensitive to Fulvestrant, as was the case with pharmacologic autophagy inhibition; however, the MCF7 shATG5 cells were only slightly more sensitive to Palbociclib and the Palbociclib + Fulvestrant combination therapy when compared to shControl cells **(** **Figures 4B–D** **)**. These data with genetic knockdown of ATG5 are consistent with the observed outcomes upon pharmacological inhibition of autophagy with CQ and Baf (**Figure 3**). Genetic silencing of autophagy also did not promote apoptosis in MCF-7 cells when exposed to Ful or Pablo, but exhibited statistically significant, albeit minimal apoptotic cell death with the combination treatment compared to shControl MCF7 cells (**Figure 4E**). Taken together, these studies indicate that the autophagy induced by the Fulvestrant, Palbociclib, and the combination treatment is largely nonprotective, and suggests the possibility that autophagy inhibition may not prove to be an effective strategy to enhance the therapeutic response (40, 42).

### 3.5 ARV-825 extends growth delay and suppresses proliferative recovery in MCF-7 breast tumor cells treated with Fulvestrant + Palbociclib

**Figure 5:**
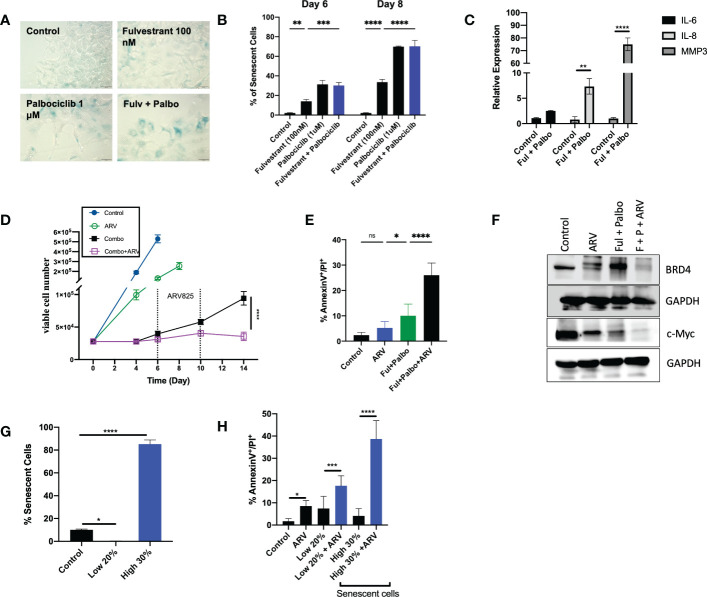
ARV prolongs growth arrest induced by Fulvestrant in combination with Palbociclib in MCF- cells. Cells were treated with Fulvestrant (100nM), Palbociclib (1uM) or the combination for 6 days. **(A)** Cells were fixed on Day 6, stained with x-gal staining solution and imaged using brightfield microscope. All images were taken with the same magnification. **(B)** Quantification of SA-Bgal using C12FDG at indicated timepoints. **(C)** qRTPCR examining SASP mRNA expression of IL-6, IL-8, and MMP3 on D6 post-combination treatment. **(D)** Cell viability was monitored over a period of 14 days by trypan blue exclusion. **(E)** Apoptosis was evaluated by flow cytometry using an APC Annexin V Apoptosis Detection Kit. **(F)** Western blotting for BRD4, c-Myc at day 4 of ARV treatment. **(G)** Sorted cells was analyzed by flow cytometry to verify purity of the SA-β-Gal positive population (high 30% and low 20% SA-β-Gal positive). Control cells were sorted for high 80% SA-β-Gal positive. **(H)** Sorted senescent cells were plated for 24-hours and treated with ARV-825 for 4 days followed by annexin V/PI apoptosis staining. Flow cytometry was performed post-sort for C12FDG staining to confirm senescence induction. *P ≤ 0.05, **P ≤ 0.01, ***P ≤ 0.001, ****P ≤ 0.0001, ns (not significant) indicate statistical significance of each condition compared to control as determined using two‐way ANOVA with Sidak's *post hoc* test. All images are representative fields, blots, or data from three independent experiments (*n* = 3).

Given that administration of Fulvestrant + Palbociclib either alone or in combination, induces a transient growth arrest, and that autophagy and senescence tend to occur in parallel, we examined senescence induction, a durable growth arrest induced by therapy (21, 29, 45–47). Previous work from our group and others has consistently shown proliferative recovery from various models of therapy induced senescence (11–16). **Figure 5A** demonstrates the promotion of senescence by β-gal staining with exposure to Fulvestrant alone, Palbociclib alone and the Fulvestrant and Palbociclib combination in MCF7 cells as well as morphological changes (cell enlargement and flattening) associated with senescence (45). Senescence was further confirmed using flow cytometry quantification of C_12_FDG fluorescence, a metabolite for SA-ß-gal. **Figure 5B** demonstrates increased SA-ß-gal activity in Fulvestrant, Palbociclib and combination treated MCF7 cells when compared to untreated controls. The senescence in the combination therapy appears to be largely due to the Palbociclib (46, 48). **Figure 5C** presents qRTPCR data indicating significant increases of the expression of IL-6, IL-8, and MMP3, components of the senescence-associated secretory phenotype (45, 49), at Day 6 post-combination treatment with Fulvestrant and Palbociclib.

We and others have demonstrated that the senolytic ABT-263 (navitoclax), is effective at the elimination of tumor cells induced into senescence by various chemotherapeutic strategies in breast, lung, head and neck and prostate tumor cells (14–16). However, one fundamental limitation in the use of ABT-263 is thrombocytopenia, given that the target of ABT-263 is the Bcl-xL protein, upon which platelets depend for survival (50).

Inhibitors of bromodomain-containing protein 4 (BRD4), particularly ARV-825, have demonstrated antitumor activity in multiple preclinical models, and have recently been considered as potential senolytics (51). To investigate whether ARV-825 might act as a senolytic in combination with the senescence induced by the Fulvestrant + Palbociclib combination, cells were treated for 6 days with Fulvestrant (100 nM) and Palbociclib (1 *µ*M), followed by ARV-825 (50 nM) for 96 h post-treatment. Temporal analysis of cell viability demonstrated that ARV-825, alone, moderately suppressed growth of the MCF-7 cells (**Figure 5D**); this is consistent with prior literature studies of the action of ARV-825 (52, 53), and with the degradation of BRD4 and the suppression of downstream c-Myc shown in **Figure 5F**. Our laboratory as well as others have shown that c-Myc is upregulated in breast cancer and involved in breast cancer proliferation (52, 54, 55).

The most critical observation in this work is that ARV-825 treatment sequentially after the Fulvestrant + Palbociclib combination resulted in a prolonged growth arrest with suppression of proliferative recovery **(** **Figure 5D** **).** This finding is consistent with the pronounced suppression of both BRD4 and c-Myc for the combination of Fulvestrant + Palbociclib and ARV-825 in **Figure 5F**.

Although the addition of ARV-825 to the Fulvestrant + Palbociclib combination appeared to result in prolongation of growth arrest rather than cell killing, this therapeutic strategy nevertheless demonstrated some characteristics of senolysis, specifically the promotion of apoptosis. As shown in **Figure 5E**, a significant increase in apoptosis was evident at Day 10 with ARV-825 treatment compared to the combination treatment alone; the percentage of apoptosis stayed relatively constant through day 14 (data not shown).

Although the combination treatment promotes substantial senescence in the MCF-7 cells (**Figures 5A, B**), the entire cell population is not senescent, and consequently it was necessary to address whether the ARV-825 was functioning as a senolytic and that the senescent cell population might be particularly vulnerable to the ARV-825. To address this question, cells were sorted by flow cytometry to distinguish the SA-β-Gal highly positive and low positive populations (high 30% and low 20% SA-β-Gal positive). Control cells were also sorted for SA-β-Gal positive cells **(** **Figure 5G** **)**. Flow cytometry was performed post-sorting for C_12_FDG staining to confirm the senescent population **(** **Figure 5G** **)**. **Figure 5H** indicates that the senescent high cell population underwent significantly more apoptosis compared to the non-senescent cells after treatment with Fulvestrant/Palbociclib + ARV-825. These data indicate that the senescence induced by the combination of an anti-estrogen and CDK4/6 inhibitor increases susceptibility to ARV-825 induced apoptotic cell death.

### 3.6 Sensitization by ARV-825 in p53 mutant T-47D breast tumor cells treated with the Fulvestrant + Palbociclib combination

**Figure 6:**
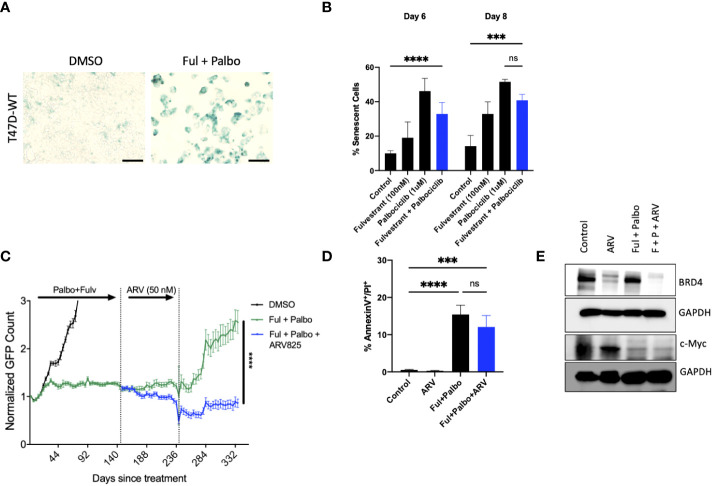
ARV prolongs growth arrest induced by Fulvestrant in combination with Palbociclib in p53 mutant T-47D cells. T47D-WT cells that were treated with palbociclib (1 µM) in combination with Fulvestrant (100 nM) for 6 days. **(A)** Cells were fixed on Day 6, stained with x-gal staining solution, and imaged using brightfield microscope. All images were taken with the same magnification. **(B)** Quantification of SA-Bgal using C12FDG at indicated timepoints. **(C)** Live cell viability was monitored *via* IncuCyte over a period of 14 days and normalized to GFP count. **(D)** Apoptosis was evaluated by flow cytometry using an APC Annexin V Apoptosis Detection Kit. **(E)** Western blotting for BRD4, c-Myc at day 4 of ARV treatment. Mean and SD were determined based on triplicates from 3 independent experiments. ***P ≤ 0.001, ****P ≤ 0.0001, ns (not significant) indicate statistical significance of each condition compared to control as determined using two‐way ANOVA with Sidak’s *post hoc* test.

Approximately 20% of ER positive breast cancers present with p53 mutations (56). In order to evaluate whether ARV-825 would also be effective against p53 mutant ER+ breast tumors treated with the Fulvestrant + Palbociclib combination, we assessed the temporal response by real time, live cell imaging using IncuCyte S3 in T-47D breast tumor cells. Analogous to the outcomes in MCF-7 cells (**Figure 5D**), we observed that the combination of Fulvestrant + Palbociclib induced senescence based on senescence associated β-galactosidase staining (**Figure 6A**) that was followed by proliferative recovery (**Figure 5C**); as in the studies with the p53 wild-type MCF-7 cells, the addition of ARV-825 resulted in prolonged growth arrest without recovery, at least over the ~ 13-day time course of this study (**Figure 6C**). Quantifying the extent of senescence using C_12_FDG staining indicated that approximately 40% of the population represented senescent cells at both day 6 and day 8 (**Figure 6B**). Analysis of apoptosis indicated that there was no significant difference with the combination + ARV825, compared to the combination alone (**Figure 6D**), despite some evidence of a decline in cell number in the temporal response study. Additionally, western blot analysis confirmed a reduction in target protein, BRD4, by ARV-825 in both control and Fulvestrant + Palbociclib (**Figure 6E**). Similarly, a profound suppression of downstream c-Myc by ARV-825 is evident in control cells, the combination alone and the combination + ARV825 (**Figure 6E**).

### 3.7 Sensitization by ARV-825 in MCF-7 cells deficient in Rb and treated with the fulvestrant + palbociclib combination

**Figure 7:**
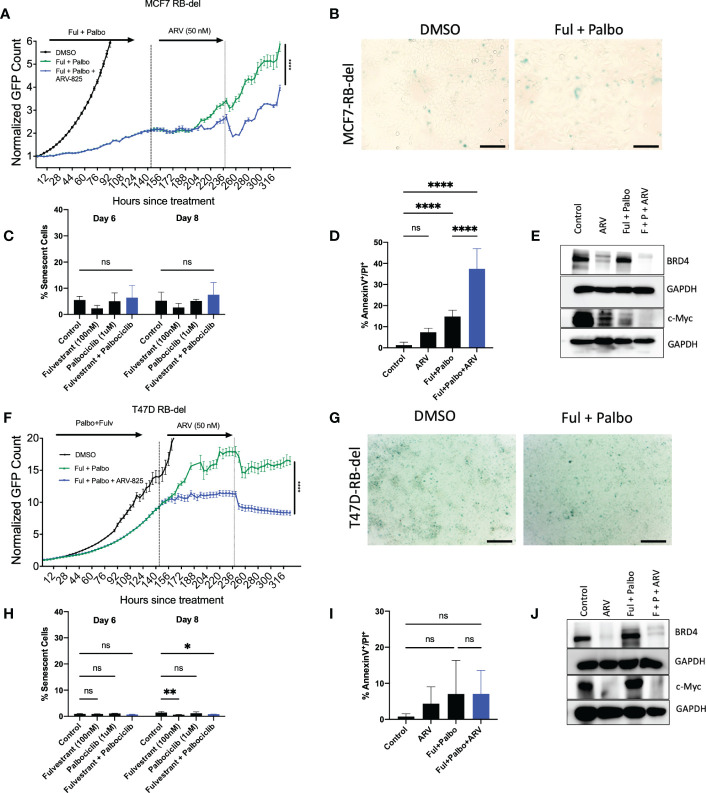
ARV-825 prolongs growth arrest induced by the combination of Fulvestrant and Palbociclib in resistant MCF-7 and T47D cells. Live cell imaging in MCF-7 Rb-del cells that were treated with palbociclib (1 µM) in combination with fulvestrant (100 nM) for 6 days. **(A)** Live cell viability of Rb-deficient MCF-7 cells was monitored via IncuCyte over a period of 14 days and normalized to GFP count. **(B)** MCF-7 Rb deleted cells were fixed on Day 6, stained with x-gal staining solution, and imaged using brightfield microscope. All images were taken with the same magnification. **(C)** Quantification of SA-Beta gal using C12FDG at indicated timepoints. **(D)** Apoptosis was evaluated by flow cytometry using an APC Annexin V Apoptosis Detection Kit. **(E)** Western blotting for BRD4, c-Myc at day 4 of ARV treatment. **(F)** Live cell viability of Rb-deficient T47D cells was monitored *via* IncuCyte over a period of 14 days and normalized to GFP count. **(G)** Cells were fixed on Day 6, stained with x-gal staining solution, and imaged using brightfield microscope. All images were taken with the same magnification. **(H)** Quantification of SA-Beta gal using C_12_FDG at indicated timepoints. **(I)** Apoptosis was evaluated by flow cytometry using an APC Annexin V Apoptosis Detection Kit. **(J)** Western blotting for BRD4, c-Myc at day 4 of ARV treatment. *P ≤ 0.05, **P ≤ 0.01, ****P ≤ 0.0001, ns (not significant) indicate statistical significance of each condition compared to control as determined using two‐way ANOVA with Sidak's post hoc test. All images are representative fields, blots, or data from two independent experiments (*n* = 2).

One common mechanism of resistance that develops to palbociclib therapy in ER positive breast tumor cells is the loss of Rb, which prevents the cells from entering into a state of senescence (57). However, it is possible that the ARV-825 is not acting as a classical senolytic, which might make cells treated with fulvestrant + palbociclib susceptible to cell killing even where senescence is not the primary response. To address this possibility, MCF-7 and T47D cells, where Rb function was genetically deleted, were exposed to the fulvestrant + palbociclib combination, followed by ARV-825. As shown in **Figure 7A**, in MCF-7 Rb deleted cells, the combination treatment promotes delayed growth compared to control, followed by proliferative recovery, similar to what we observe with Rb competent MCF-7 cells. **Figure 7B** demonstrates the lack of senescence induction with MCF-7 Rb deleted cells, which was confirmed with quantification of senescence using C_12_FDG where we observe a relatively low percentage of senescent cells **(** **Figure 7C** **)**. The addition of ARV-825 to the combination treatment of fulvestrant + palbociclib clearly promotes an increase in apoptosis over the low level of apoptosis induced by fulvestrant + palbociclib **(** **Figure 7D** **)**. **Figure 7E** indicates that ARV-825 suppressed both the target protein, BRD4, as well c-Myc, which is presumably downstream of BRD4, in both control and fulvestrant + palbociclib treated cells.

In contrast to the MCF-7/Rb deleted cells, growth arrest by the treatment with the fulvestrant + palbociclib combination is markedly delayed in the T47D Rb deleted cells; nevertheless, the addition of ARV-825 significantly suppresses growth **(** **Figure 7F** **)**. As was largely the case with the MCF-7/Rb deleted cells, the combination treatment fails to promote senescence (**Figure 7G**), as further confirmed by C_12_FDG quantification **(** **Figure 7H** **)**. Furthermore, while MCF-7 Rb deficient cells demonstrated enhanced apoptosis with the combination treatment followed by ARV-825 **(** **Figure 7D** **)**, there was no increase in apoptosis in the T47D Rb deficient cells **(** **Figure 7I** **)**. This is likely related to the absence of functional p53, as there was also no increase in apoptosis in the Rb proficient T-47D cells (**Figure 6D**). Finally, the targeting of BRD4 and c-Myc by the ARV-825 was confirmed by the Western blot presented in **Figure 7J**.

## 4 Discussion

Disease recurrence, both local and distal, is an ongoing issue contributing to the majority of hormone receptor-positive breast cancer deaths and is observed in many other types of cancers, such as triple-negative breast cancer, lung, and prostate cancer. Often this recurrence can be associated with therapy-induced residual dormant tumor cell populations, that can escape and often become more aggressive in nature (58–60). While therapy-induced senescence has been studied for decades, successful utilization of senolytics in cancer treatment has not yet been implemented. Despite this, several senolytic agents have been considered and studied to modulate and eliminate senescent tumor cells.

Another potential route for modulation of drug sensitivity in cancer is autophagy inhibition (23, 61). Estrogen receptor-targeted therapies are generally the first-line treatment for hormone receptor-positive breast cancer and autophagy in response to tamoxifen has been shown quite convincingly to be cytoprotective (62). In fact, this form of autophagy has been shown to lead to the development of resistance to anti-estrogen therapies (44, 63, 64). In cases where autophagy is cytoprotective, pharmacological inhibition of autophagy may be utilized to enhance the tumor cell sensitivity to treatment. In addition to cytoprotective autophagy, we have identified a non-protective form of autophagy, which apparently plays no distinct role in promoting or suppressing the growth or sensitivity of the tumor cells in response to therapy (39, 40, 61). Clinical trials are currently underway evaluating HCQ as a pre-treatment with the combination of letrozole + Palbociclib, based on preclinical studies showing efficacy in autophagy inhibition as a pretreatment with this combination (25).

The current work evaluated the potential of utilizing autophagy inhibition, before and after treatment, to sensitize ER positive MCF-7 breast tumor cells to the combination of Fulvestrant + Palbociclib. This treatment promotes significant growth arrest and both autophagy and senescence. induction after 6 days of treatment with Fulvestrant and Palbociclib. Early administration of pharmacological autophagy inhibitors did not improve tumor cell responsiveness to the combination treatment, leading to the conclusion that the autophagy was non-protective. Slight sensitization was evident with late addition of pharmacological autophagy inhibitors as well as with genetic knockdown. The latter was most pronounced with Fulvestrant, which may relate to previous studies demonstrating that ER targeted therapies are generally cytoprotective (65, 66). However, the autophagy for the combination treatment appears to be largely nonprotective, indicating that autophagy inhibition is unlikely to become a clinically useful therapeutic strategy (61, 67). This does not rule out the possibility that the autophagy induced by aromatase inhibitors in combination with cdk4/6 inhibitors could be cytoprotective and amenable to autophagy inhibition in the clinic.

Our studies further examined the incorporation of a BET degrader, ARV-825, into the combination treatment. We initially screened a variety of agents from different drug classes that had been reported to have senolytic activity and found that this BET inhibitor was the most promising agent. Although we had published previous studies using ABT-263 (navitoclax), we chose not to continue with this class of drugs due to the thrombocytopenia associated with the administration of Bcl-xL targeted agents. BET inhibition has demonstrated efficacy in many clinical trials consisting of both hematological malignancies as well as solid tumors (68). BET degrader ARV-825 has been used in pre-clinical studies with different cancer types, and we hypothesized that ARV-825 could potentially improve ER+ breast cancer tumor response following Fulvestrant + Palbociclib treatment (59–62). ARV-825 suppressed tumor growth for both the ER positive p53 wt MCF-7 cells and the ER positive p53 mutant T-47D cells, significantly delaying proliferative recovery. We also observed significant induction of apoptosis in the MCF-7 cells treated with Fulvestrant + Palbociclib followed by ARV-825, but not in T47D cells, which may be due to T-47D p53 mutational status. The senescent MCF-7 cell population appears to be more susceptible to ARV-825 induced apoptosis, although a low degree of apoptosis is also observed in non-senescent cells exposed to the ARV-825. While the combination of Fulvestrant + Palbociclib rapidly induced growth arrest in MCF-7 Rb deficient cells, the growth was delayed in the T47D Rb deficient cells. The observed growth arrest was confirmed to be independent of senescence, as Rb loss is a well-known factor of resistance to CDK 4/6 inhibitors (69, 70).

The observed growth arrest is consistent with the degradation of BRD4 and the suppression of downstream c-Myc as well as with previous studies by our laboratory and others demonstrating c-Myc to be upregulated in ER+ breast cancer and involved in breast cancer proliferation (52, 54, 55). These findings are supported by an analysis of three patient database sets, demonstrating that high expression of BRD4 that can be observed across multiple subtypes of breast cancer is correlated with overall lower recurrence-free survival when compared to patients with low BRD4 expression levels (71).

Taken together, the current studies indicate that administration of BET inhibitors/degraders may potentially improve the standard of care therapy in metastatic ER+ breast cancer patients and may further prolong progression-free survival. Further validation of these findings in cell culture will require studies in tumor-bearing animals. The BET inhibitors, ABBV-075 and ABBV-744, have shown promise in preclinical studies and will be tested in the future both in cell culture and in tumor-bearing animal models (72–74).

## Data availability statement

The original contributions presented in the study are included in the article/**Supplementary Material**. Further inquiries can be directed to the corresponding authors.

## Author contributions

RF, LM, DG contributed to conception and design of the study. RF, AE, NP, TT, VK contributed to methodology. RF contributed to data curation. RF wrote the first draft of the manuscript. RF, NP, VK wrote sections of the manuscript. All authors contributed to the article and approved the submitted version. Research in Dr. Gewirtz’s laboratory is supported by grants # CA268819 and CA239706 from the National Cancer Institute/National Institutes of Health and Grant # W81XWH 19-1-0490 from the Department of Defense Congressionally Directed Breast Cancer Research Program.

## Supplementary material

Supplementary Figure 1 Autophagy inhibition does not alter sensitivity to Fulvestrant in combination with Palbociclib in T47D cells. Cells were pre-treated for 3 h with either CQ (10 uM) or Baf A1 (2.5 nM). CQ and Baf A1 were given for an additional 48 h, alongside the treatment with Fulvestrant (100 nM), Palbociclib (1 µM) or the combination for 6 days. **(A)** Schematic of *in vitro* treatment of early addition of CQ and Baf. **(B)** Percent cell viability was measured using MTS viability assay. **(C)** Western blot analysis at day 6 assessing accumulation of LC3 I-II. **(D)** Schematic of *in vitro* treatment of late addition of CQ and Baf. **(E)** Percent cell viability was measured using MTS viability assay. **(F)** Western blot analysis at day 6 assessing accumulation of LC3 I-II.
