# Supplementary figures and images for: The BET inhibitor/degrader ARV-825 prolongs the growth arrest response to Fulvestrant + Palbociclib and suppresses proliferative recovery in ER-positive breast cancer
Source: Front Oncol. 2023 Jan 18;12:966441. doi: 10.3389/fonc.2022.966441 (PMC9890056; doi:10.3389/fonc.2022.966441)

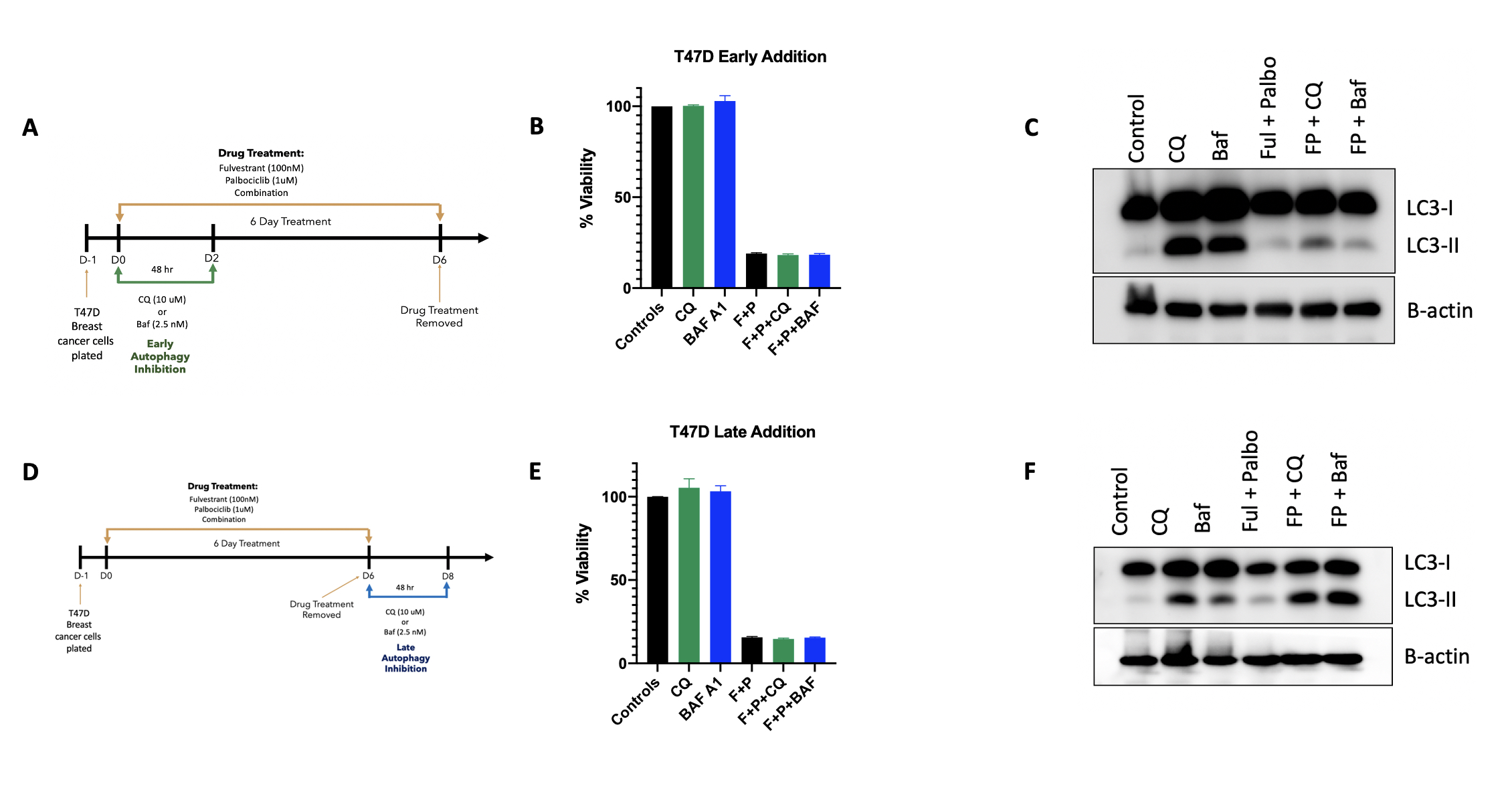

Supplement: Supplementary Figure 1 — Autophagy inhibition does not alter sensitivity to Fulvestrant in combination with Palbociclib in T47D cells. Cells were pre-treated for 3 h with either CQ (10 uM) or Baf A1 (2.5 nM). CQ and Baf A1 were given for an additional 48 h, alongside the treatment with Fulvestrant (100 nM), Palbociclib (1 µM) or the combination for 6 days. (A) Schematic of in vitro treatment of early addition of CQ and Baf. (B) Percent cell viability was measured using MTS viability assay. (C) Western blot analysis at day 6 assessing accumulation of LC3 I-II. (D) Schematic of in vitro treatment of late addition of CQ and Baf. (E) Percent cell viability was measured using MTS viability assay. (F) Western blot analysis at day 6 assessing accumulation of LC3 I-II. [file Image_1.jpg]
